# Hospitals for the Middle Classes

**Published:** 1924-02

**Authors:** Edith Johns

**Affiliations:** Lewisham Hospital, S.E. 13


### Hospitals for the Middle Classes.

SIR,—In the January issue of the HOSPITAL AND HEALTH REVIEW appeared an article entitled " Hospitals for the Middle Classes." The following extract interested me very deeply : " It [the suggested hospital] should provide a Training School for Nurses, for the employment of trained nurses only, while not increasing efficiency, would raise unduly the cost of nursing."

Herein, I contend, lies the whole difficulty of the progress of nursing as a profession. The primary object of a training school for nurses is, it would seem, not to supply the country with efficient trained nurses, or to raise the status of nursing as a profession, but merely to keep down the cost of the nursing staff ! I feel convinced that the greatest need of the nursing profession at the moment is to widen the channels of the outflow rather than of the source. Wider opportunities for the nurses when they are trained are of the greatest importance. It makes one wonder what becomes of the numbers of trained nurses who are turned out of the training schools every year. How few are the posts open to them, and, in most cases, the pitifulness of the salaries offered ! Salaries, of course, are purely a question of supply and demand. It would seem that a Central Body for the control of the supply of Probationers, interested in the welfare of the nursing staffs as well as of the hospitals, is certainly needed.

EDITH JOHNS.

Lewisham Hospital, S.E. 13.

